# Adolph Seeligmüller (1837–1912)

**DOI:** 10.1007/s00415-020-10268-w

**Published:** 2020-10-21

**Authors:** Carolin Arendt, Stephan Zierz

**Affiliations:** Department of Neurology, Neurologische Universitätsklinik Martin-Luther-Universität Halle-Wittenberg, Ernst-Grube-Str. 40, 06120 Halle (Saale), Germany

Adolph Seeligmüller (Fig. 1), born on April 1, 1837 in Naumburg (Saale) [1], was a neurologist in Halle (Saale). According to *Adams and Victor’s Principles of Neurology (Ninth Edition)*, he was the first to describe hereditary spastic paraplegia in 1874. In addition, Alma Kreuter, in her classical book on German-speaking neurologists and psychiatrists, mentioned Seeligmüller as the first author on myotonia congenita before Julius Thomsen [2].

**Fig. 1:**
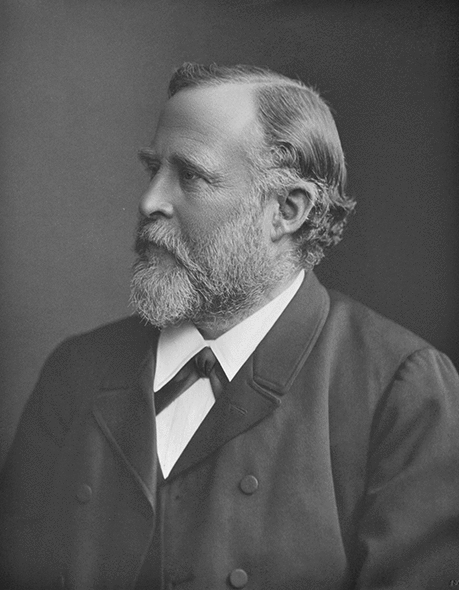
Adolph Seeligmüller, a neurologist from Halle and a teaching professor of nervous diseases and nerve pathology at the former “Vereinigte Friedrichs-Universität Halle-Wittenberg” (today known as Martin-Luther-University Halle-Wittenberg) [University Archive Martin-Luther-University Halle-Wittenberg: UA Halle, Rep. 40-VI, Nr. 1, Bild 041]

After studying medicine, psychology, natural sciences, and philosophy in Halle, Leipzig, and Würzburg [2, 3], Seeligmüller visited Vienna and Paris, where he worked with Benedict and Duchenne [2, 3]. His doctoral thesis (Halle/Saale 1861) was *De tumoribus cavi mediastini solidis, praecipue carcinomatosis* [1–3]. As an assistant physician, he worked in the provincial asylum in Nietleben near Halle and later in the department of Internal Medicine of the University of Halle [1, 3]. In 1865 he started to work in his private neurological practice in Halle [3]. His “habilitation” (Halle/Saale, 1876) was *De Nervi symathici laesionibus traumaticis* [1, 3].

Seeligmüller headed the neurological “Poliklinik” (outpatient department) of the University of Halle from 1876 until 1904 [2, 3]. As an associate professor, he lectured 26 years neurology at the University in Halle [1]. The topics of his lectures also included hypnotism originating from mesmerism (animalic magnetism) [1].

Beginning from about 1890, Seeligmüller had some quarrels with the medical faculty and the dean Eduard Hitzig (1838–1907), who was at the same time head of the royal psychiatric and nerve clinic in Halle. The origins of this conflict were discussions on the differentiation between posttraumatic neuroses and simulation. Seeligmüller proposed establishing special hospitals (*Unfallkrankenhäuser*) to differentiate between posttraumatic and simulated symptoms by appropriately trained and experienced doctors [4]. Hitzig was particulary bothered, because Seeligmüller called the university clinics "universities of simulation" [4] These quarrels could have been the real reason why Seeligmüller left the neurological “Poliklinik” of the university for private practice in 1904 and finally also requested the suspension from his teaching duties at the university in 1907 [1–3].

On April 19, 1912, Adolph Seeligmüller died in Halle at the age of 75 years [1]. In his funeral eulogy, Gabriel Anton (1858–1933) noted that Seeligmüller “was one of the first in the difficult field of spinal cord diseases” [1], and acknowledged Seeligmüller’s most outstanding work, his *Lehrbuch der Krankheiten der peripheren Nerven und des Sympathikus (1882)*, and his *Lehrbuch der Krankheiten des Rückenmarks und des Gehirns und der allgemeinen Neurosen* (1887) [5]. Seeligmüller summarized the state of neurology at that time, not only for students but also for colleagues “who did not have the opportunity to learn neurology so extensively during their studies” [5]. In the introduction to the second volume, he stated that both volumes were the “most complete textbook on neurology without including psychiatric diseases” [5]. Seeligmüller tried to link anatomy and physiology to the pathologies and diseases [5]. Remarkably, Seeligmüller´s both textbooks appeared shortly before Gowers seminal *Diseases of the nervous system* in 1886 and 1888.

In addition to this textbook, Seeligmüller also published a vast number of studies. A syphilis-based neuralgia of the nervi auriculotemporalis et occipitalis minor was named Seeligmüller’s neuralgia [2]. Alma Kreuter provides a comprehensive overview of his more than 70 publications on various topics of peripheral and central nervous system diseases [2].

In 1876, Seeligmüller described a sclerosis of the lateral corticospinal tract in four children born from a consanguineous marriage [6]. As mentioned above, this is supposed to be the first description of HSP. Seeligmüller himself suspected that he described an infantile form of lateral sclerosis (corresponding to Charcot’s lateral sclerosis in adults). The symptoms of these children were similar to a symptom complex first described by Wilhelm Erb in 1875 (*Erb W. Ueber die spastische Spinalparalyse, 1877*). However, other signs such as muscle atrophy and involvement of the cranial nerves (bulbar paralysis) mentioned by Seeligmüller were not part of the symptom complex described by Erb. The symptom complex described by Erb was also observed in patients by Adolf Strümpell (*Strümpell A. Beiträge zur Pathologie des Rückenmarks. I. Spastische Spinalparalyse, 1880*). In 1893, Strümpell first introduced the term “hereditary spastic spinal paralysis” [7]. Thus, the merit of the first description of HSP symptoms belongs to Erb (and Charcot in France 1876). However, Strümpell (and Lorrain in France 1898) recognized the hereditary nature and identified the pathological basis. Thus, the notion that Seeligmüller first described HSP is not warranted because muscle atrophy and bulbar involvement are not typical for HSP.

In 1876, myotonia congenita was described both by Julius Thomsen (1815–1896), at that time “Kreisphysikus” (district physician) in Kappeln [8], and by Adolph Seeligmüller [9]. However, the term “myotonia congenita” was first proposed by Adolf Strümpell in 1881 [10]. Seeligmüller’s article appeared on August 19, 1876, shortly after Thomsen. Thomsen published detailed hypotheses on symptoms, pathology, etiology, and therapy for a hitherto unknown disease entity, which he and parts of his family suffered from since early childhood [8]. Thomsen, Seeligmüller and Strümpell mentioned earlier descriptions of similar symptoms by Bell, Benedict, and von Leyden [8–10]. The publication by Thomsen was prompted because his son, who was affected with the disease, was accused of malingering to avoid military duty. More than 1 year earlier, Seeligmüller had also examined a patient whose symptoms matched exactly with the disease described by Thomsen [9].

Despite differing views on interpretation as ataxia, and pathological localization, both articles described what is now known as myotonia congenita. Already Strümpell recognized that Thomsen’s paper suffered “from a certain subjectivity” and that Thomsen addressed the psyche a “strong influence” [10], while Seeligmüller emphasized the neuromuscular aspect [9].

In summary, Seeligmüller was not the first to describe HSP but described myotonia congenita shortly after Thomsen in the same year. Because of this, his seminal textbook on neurology and his numerous clinical publications on a wide spectrum of neurological diseases, Seeligmüller certainly belongs to the great neurologists of the nineteenth century.

## Appendix

**Table:** 

Name	Location	Role	Contribution
Carolin Arendt	Department of Neurology, University of Halle/Saale	Author	Literature research and analysis, drafting of manuscript
Stephan Zierz, MD	Department of Neurology, University of Halle/Saale	Author	Discussion and revision of manuscript

## Data Availability

We take full responsibility for the data, the analyses and interpretation, and the conduct of the research, and have full access to all of the data.
